# Fast shape memory function and personalized PLTMC/SIM/MBG composite scaffold for bone regeneration

**DOI:** 10.1016/j.mtbio.2025.101791

**Published:** 2025-05-02

**Authors:** Xulin Hu, Shengwen Cheng, Senrui Liu, Minchang Zhou, Junyan Liu, Jiaying Wei, Yixuan Lan, Yu Zhai, Xiaohong Luo, Mingfei Dong, Zu Xiong, Wei Huang, Chen Zhao

**Affiliations:** a Clinical Medical College & Affiliated Hospital of Chengdu University, Chengdu University, Chengdu, 610081, PR China; b Department of Orthopaedic Surgery, The First Affiliated Hospital of Chongqing Medical University, Chongqing, 400016, PR China; c Chongqing Municipal Health Commission Key Laboratory of Musculoskeletal Regeneration and Translational Medicine, 400016, Chongqing, PR China; d Orthopaedic Research Laboratory of Chongqing Medical University, Chongqing, 400016, PR China

**Keywords:** Bone defect repair, Porous composite scaffold, 3D low-temperature rapid prototyping, Mesoporous bioactive glass, poly(L-lactide-co-trimethylene carbonate)

## Abstract

Orthopedic clinical practice faces significant challenges in treating critical-sized bone defects due to extensive tissue damage and prolonged healing. To address these limitations, this study integrated shape-memory polymers with 3D printing to engineer bioactive scaffolds composed of poly(l-lactide-co-trimethylene carbonate) (PLTMC), simvastatin (SIM), and mesoporous bioactive glass (MBG) via low-temperature rapid prototyping. The PLTMC/SIM/MBG composite scaffold exhibited exceptional porosity (78.5 % ± 1.5 %) and load-bearing compressive strength (66.33 ± 1.44 MPa at 30 % MBG). In addition, its thermoresponsive shape-memory behavior enabled intraoperative molding to precisely conform to defect geometries, while the sustained release of SIM and MBG ionic exchange together created a bioactive microenvironment. Mechanistically, the scaffold activated the Wnt pathway to enhance the osteogenic differentiation of mesenchymal stem cells, maintaining cytocompatibility. In vivo, directional bone regeneration occurred along the degradable scaffold, driven by synergistic topographical guidance from 3D-printed pores and biochemical cues from SIM and MBG. The shape-adaptive design preserved mechanical continuity with the host bone during remodeling. These results demonstrate a personalized solution for large defects, merging surgical adaptability through shape-memory functionality with bioactive efficacy via structural and biochemical synergy, overcoming the limitations of conventional implants in anatomical matching and regenerative performance.

## Graphical abstract

Inspired by the concept of designing functional materials, we have innovatively fabricated a novel PLTMC/SIM/MBG composite scaffold via low - temperature 3D printing technology, endowing it with body temperature - responsive shape memory functionality. This cutting - edge scaffold adopts a multi - pronged strategy to promote bone regeneration: the mesopores of MBG enable the controlled release of SIM and facilitate Ca²⁺/SiO₄⁴⁻ ion exchange; its optimized porosity of 78.5 % and compressive strength of 66.33 MPa are tailored to meet the mechanical demands of cancellous bone; activation of the Wnt/β - catenin pathway effectively promotes the osteogenic differentiation of BMSCs; and in the rat femoral defect model, it showcases remarkable performance in achieving orderly bone regeneration and vascularization, embodying the practical application.

**Figure:**
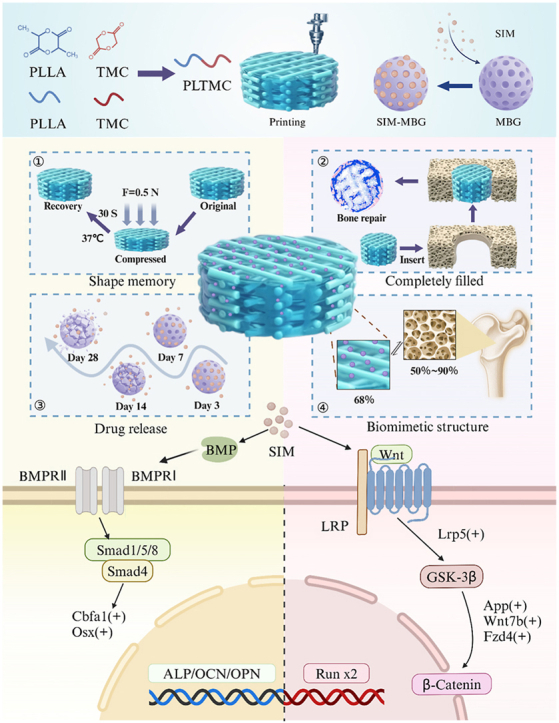

## 1 Introduction

Bone defects resulting from traumatic injuries, tumor resections, or osteomyelitis debridement [1,2] represent a major clinical burden, annually affecting over 20 million patients around the globe, with treatment costs exceeding USD 5000 per case [3,4]. Traditional repair methods like autografts and allografts are constrained by donor-site morbidity, immune rejection risks, and limited supply [5,6], thus necessitating innovative alternatives [7]. Bone tissue engineering (BTE) has emerged as a transformative approach, offering personalized solutions through anatomically tailored scaffolds fabricated via 3D printing technology [8,9]. These customized constructs enable precise defect matching while enhancing host tissue integration. However, critical challenges persist in optimizing scaffold materials and refining printing techniques, particularly factors determining the structural integrity, bioactivity, and clinical feasibility of 3D-printed bone substitutes [10]. Current limitations in material selection, including balancing biodegradability with mechanical stability and ensuring osteoconductive properties, hinder the replication of the hierarchical architecture of native bones. Simultaneously, technical constraints in 3D printing resolution and scalability complicate the translation of patient-specific designs into functional implants. Addressing these dual challenges in material innovation and advanced manufacturing is pivotal to unlocking the full potential of BTE for complex bone defect repair, bridging the gap between anatomical customization and biologically active regeneration.

The development of stimuli-responsive shape-memory biomaterials has revolutionized bone repair by enabling dynamic adaptation to defect geometries and personalized therapeutic strategies [[11], [12], [13]]. Although these materials offer programmable shape recovery under specific conditions (e.g., body temperature) and customizable designs, critical limitations persist. Traditional shape-memory polymers often exhibit inadequate biodegradability, mechanical mismatch with natural bone, and activation temperatures misaligned with physiological ranges, compromising their clinical utility in load-bearing applications [14,15]. To address these challenges, we engineered poly (l-lactide-co-trimethylene carbonate) (PLTMC) scaffolds via 3D low-temperature rapid prototyping (LT-RP), optimizing the lactic acid/trimethylene carbonate (LA/TMC) monomer ratio to achieve a glass transition temperature (Tg) of 25–37 °C [[16], [17], [18]]. This strategy can be achieved through preoperative computed Tomography (CT) modeling and stent printing, followed by compression implantation using a shape-memory function that restores the printed state at body temperature [19]. This process avoids the need for intraoperative adjustments when using nonconformable stents, and it reduces secondary injuries to patients. Furthermore, this strategy introduces TMC segments, which significantly reduce the acidic degradation products typically produced by pure polylactic acid [20]. It also enables surface dissolution degradation, similar to PLTMC, allowing the implanted stent to maintain its overall shape while gradually regressing inward during the degradation process. However, most biodegradable synthetic polymers exhibit poor processing performance because they are prone to degradation at prolonged high temperatures, resulting in a significant loss of mechanical properties. As a solution, 3D LT-RP has been proposed as a novel prototyping technique that prevents both the thermal degradation of biodegradable synthetic polymers and the deactivation of encapsulated active substances or drugs at high temperatures [21]. For example, cucurbitacin B (CuB) has been integrated into a porous PLGA/β-TCP scaffold, which was used to enhance the osteogenic differentiation of stem cells [22], thereby demonstrating the potential of a shape-memory biomaterial in combination with 3D LT-RP technology as a regenerative therapeutic drug-delivery platform. It was found that this printing method could regulate secondary pores by adjusting the ink ratio and freeze-drying conditions, further mimicking the structure of bone grafts.

Osteogenic regulation through targeted pharmacological activation presents a critical strategy for bone repair, with simvastatin (SIM) emerging as a promising candidate because of its dual osteogenic and angiogenic properties [23]. SIM enhances bone formation by upregulating BMP2 expression and suppressing osteoclast activity while promoting angiogenesis via vascular growth factor secretion [24,25]. However, administering it systemically can be problematic because of its poor bioavailability at defect sites due to hepatic first-pass metabolism, thus necessitating high doses that risk hepatotoxicity, rhabdomyolysis, and localized inflammation [26,27]. To address these limitations, we leveraged mesoporous bioactive glass (MBG)-integrated scaffolds for localized SIM delivery. The hierarchical mesoporous structure of MBG enables high drug-loading efficiency, while its surface erosion properties synergize with scaffold degradation kinetics to achieve sustained, spatiotemporally controlled SIM release [28,29]. This approach minimizes systemic exposure by confining therapeutic concentrations to the defect microenvironment, thus circumventing dose-dependent toxicity. Furthermore, the inherent ion-exchange capability of MBG enriches the local milieu with osteogenic ions (e.g., Ca2+, SiO44−), amplifying the bioactivity of SIM through biochemical synergy. By integrating the drug-delivery precision of MBG with the pleiotropic mechanisms of SIM, this strategy overcomes the pharmacological and pharmacokinetic barriers of conventional SIM administration, enabling localized therapeutic efficacy while mitigating systemic risks. This dual-functional system exemplifies a paradigm shift in bone defect repair, merging material-driven drug delivery with targeted molecular regulation to optimize regenerative outcomes.

**Fig. 1:**
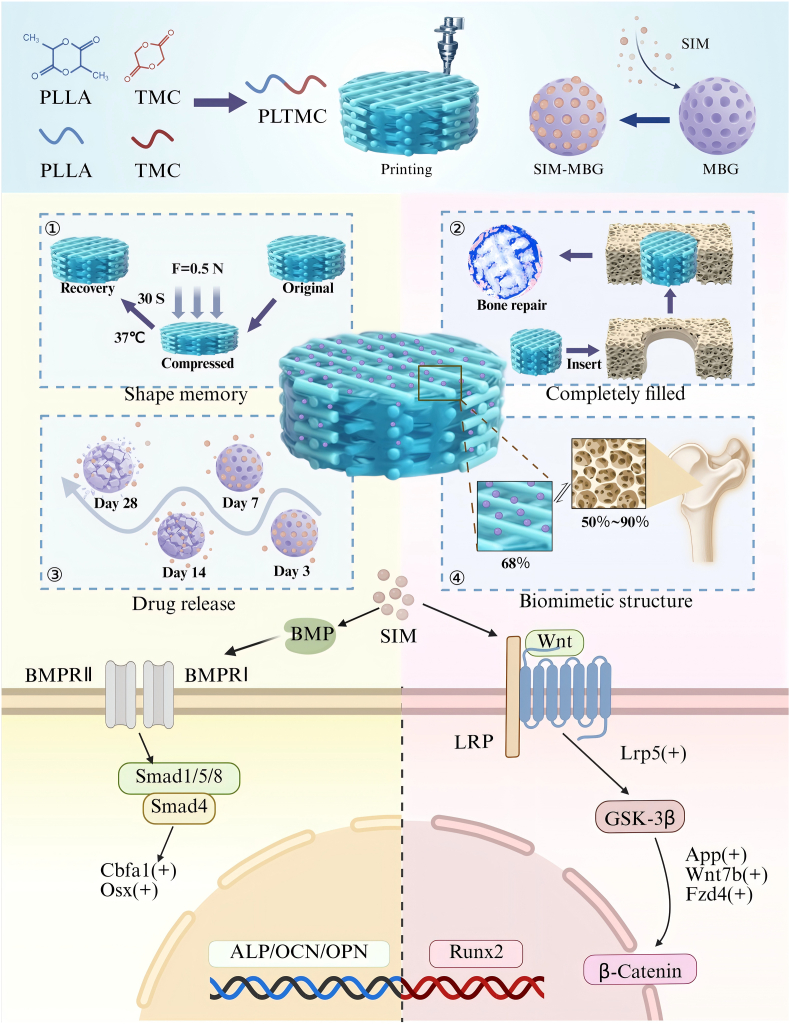
Schematic illustration of preparation of SIM/PTM scaffold and its mechanism for bone defect repair.

Given the excellent performance of shape-memory polymers, we designed multifunctional shape-memory-polymer-based bone repair scaffolds that meet the requirements of critical bone defect repairs that are incapable of self-healing. We previously designed shape-memory materials with relatively suitable shape-recovery rates at physiological temperature (37 °C). The neutral environment and surface dissolution mode of the material during degradation are expected to provide a platform for the sustained release of drugs while providing the desired mechanical properties. Although these materials exhibit good physical properties, their ability to induce osteogenesis is limited. Therefore, the addition of SIM to a bone repair scaffold to increase the osteogenicity of the material is expected to leverage the advantages of both the drug and the material, thereby providing a comprehensive BTE solution. With these considerations, in the present study, we designed MBG as a drug vehicle and used the LT-RP 3D printing technology to prepare a composite scaffold with a hierarchical pore structure composed of MBG-embedded PLTMC loaded with SIM. Fig. 1 depicts the preparation of the PLTMC/SIM/MBG scaffold and its bone defect repair mechanism. This scaffold provides a multifunctional platform that not only stimulates bone formation but also provides an environment conducive to integrating newly formed bone with its surrounding tissue. The shape-memory properties of the scaffold facilitate easy manipulation and precise placement at the defect site, thereby ensuring optimal contact with the local environment for effective drug delivery. This approach combines the biological activity of SIM with the controlled release capabilities of MBG and the mechanical advantages of shape-memory polymers. This combination effectively promotes the osteogenic differentiation of BMSCs and accelerates bone defect repair by activating the Wnt signaling pathway, thereby providing a comprehensive BTE solution.

## 2 Materials and methods

### 2.1 Materials

PLTMC was provided by the Chengdu Institute of Organic Chemistry, Chinese Academy of Sciences.

### 2.2 Preparation of SIM doped mesoporous bioactive glass

**Fig. 2:**
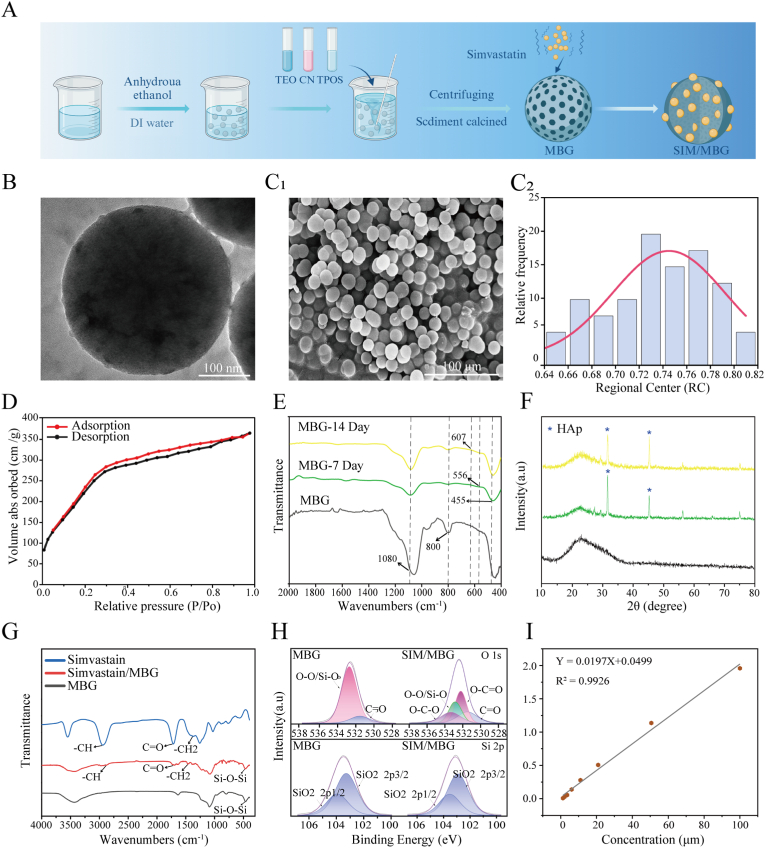
Characterization of the structural features of SIM/MBG particles. (A) Flow chart of MBG synthesis. (B) TEM image of MBG particles. (C) SEM image and particle size analysis of MBG particles. (D) N_2_ adsorption–desorption isotherms of MBG particles. (E) FTIR spectra of MBG after mineralization. (F) XRD of MBG after mineralization. (G) FTIR spectra of MBG after SIM loading. (H) XPS of MBG after SIM loading. (I) Standard curve of SIM.

MBG was prepared using a method previously described, with certain modifications [30]. Details of the method used to synthesize the SIM/MBG bone-graft substitutes are provided in Fig. 2A. Initially, a specific proportion of deionized water and anhydrous ethanol were mixed in a beaker, and an appropriate amount of CTAB was added and stirred for 30 min. Then, the ammonia solution was added slowly, and the mixture was stirred continuously for 15 min. Next, tetraethyl orthosilicate (TEOS), triethyl phosphate (TEP), and calcium nitrate tetrahydrate (CN) were added in sequence. After stirring the mixture for 5.5 h, the precipitate was separated by centrifugation and washed repeatedly to remove the solvent. Subsequently, the precipitate was dried and calcined at 650 °C in a muffle furnace to obtain MBG samples. To load the drug, 1g of MBG was mixed with 20, 50, 100, and 200 μM of SIM in 10 mL of deionized water and stirred for 4 h. The solid was then filtered and air-dried at room temperature to yield SIM/MBG. In addition, a solution containing 100 μM SIM was initially prepared and then diluted proportionally to provide 0.1, 1, 2.5, 5, 10, 20, 50, and 100 μM standard solutions, with deionized water used as the blank control. The relationship between concentration and absorbance was determined at 238 nm, and a concentration standard curve was constructed to provide a standard equation for SIM (Fig. 2I).

### 2.3 Preparation of PLTMC

Synthesis of PLTMC via Open-Loop Polymerization: PLLA and TMC monomers were mixed in a mass ratio of 70:30, then stannous octoate (0.05 wt%) was added and placed in a reaction flask. The mixture was vacuum-dried for 1 h and subsequently subjected to a polymerization reaction at 140 °C to generate the product. After 48 h, the product was completely dissolved in dichloromethane and gradually added dropwise to 6 times its volume of anhydrous ethanol to precipitate the purified product. The product was then placed in a vacuum oven and dried at 37 °C to obtain the purified material. Gel permeation chromatography (GPC) was used to determine the copolymer's molecular weight, with calibration performed using polystyrene standards. The glass transition temperature (Tg) of the PLTMC material was measured by Differential Scanning Calorimetry (DSC), using indium as the calibration standard and a sealed empty aluminum disk as the reference. A sample weighing 5–8 mg was placed in a sealed aluminum dish for testing.

### 2.4 D printing of PLTMC/MBG (PTM) scaffolds

Firstly, the printing layer height was set to 0.41 cm, the number of layers was set to 12, the line spacing was set to 0.9 mm, and the printing size was set to a cube of (1.0) cm × (1.0) cm × 0.4 cm by using printing software. Next, PLTMC was dissolved in dioxane, and 10 %, 20 %, and 30 % (w/w) of SIM/MBG were added, with an overall solid-liquid ratio of 1:8. Then, ultrasound treatment and continuous magnetic stirring were applied to ensure the uniform dispersion of MBG particles, and thereby the bioink was formed. The bioink was loaded into a 20 mL cartridge, the material was extruded through a 0.41 mm nozzle under a pneumatic pressure of 20 kPa, and it was printed at a rate of 350 mm/min. Concurrently, the material cylinder was maintained at 25 °C and the printing substrate was maintained at −5 °C. After the scaffold was printed layer by layer, it was removed and immediately placed in a vacuum freeze dryer at −70 °C for pre-freezing for 2 h. Next, the pre-frozen scaffold was transferred to freeze-drying equipment for a 48-h freeze-drying process. Finally, the freeze-dried scaffold was removed and placed in a 37 °C blast oven for 72 h to completely remove the solvent inside the scaffold.

### 2.5 Characterization of the morphology of MBG powders and PTM scaffolds

The material's morphology and composition were examined using the following analytical techniques: scanning electron microscope (ApreoC, ThermoFisher, USA); transmission electron microscope (JEM-1200EX, Japan); N2 adsorption-desorption test (ASAP 2460); Fourier transform infrared spectroscopy (FT-IR -650S, China), input voltage 100V–240V, frequency 47–63Hz, input power 42W; X-ray diffraction (XRD, DX-2700BH, China); differential scanning calorimetry (DSC, METTLER TOLEDO, Switzerland); ultraviolet–visible spectroscopy (UV–Vis, UV-5300, METASH, China); energy dispersive X-ray spectroscopy (EDS, ELECT SUPER, EDAX). Additionally, pH monitoring experiments were conducted in phosphate-buffered saline (pH 7.4) to simulate physiological conditions.

### 2.6 In vitro experiment

To evaluate the effect of PTM scaffold materials on cellular activity, a 1 cm × 1 cm × 0.4 cm sample was immersed in medium for 72 h. Mouse mesenchymal stem cells (MSCs) were then seeded in 6-well plates and cultured in medium supplemented with scaffold-soaked DMEM. Over a 7-day period, live/dead staining was conducted at various time points, and cells were observed using Nikon fluorescence microscopy, where live cells fluoresced green and dead cells appeared red. Cell proliferation was assessed using the Cell Counting Kit-8 (CCK-8) assay at days 1, 3, 5, and 7, with absorbance measured at 450 nm. BMSCs were seeded at a density of 2 × 104 cells per well in 24-well plates, and cultured with bone replacement medium and extracts from each scaffold group. Alkaline phosphatase (ALP) and alizarin red staining were both performed on days 7, 14, and 21. Total RNA was extracted for real-time PCR analysis, with primer sequences provided in Table S1. RNA sequencing was also performed to analyze the mRNA expression profile of BMSCs cultured with scaffold extracts for 7 days, using blank osteogenic medium as the control.

### 2.7 In vivo experiment

Male rats (350–450 g) were sourced from the Chongqing Medical University Laboratory Animal Center. Anesthesia was induced by administering pentobarbital at 40 mg/kg. Under sterile conditions, a 4 mm diameter, 5 mm deep cylindrical bone defect was created in the femoral condyle of each rat femur. A total of 40 rats were randomly assigned to four groups: a control group (no material), a material control group (PTM), an experimental group (20 SIM/PTM), and an experimental group (100 SIM/PTM). In vivo bone regeneration was assessed using high-resolution micro-computed tomography (microCT; Scanco micro-CT100, Switzerland). A 4 mm diameter region of interest (ROI) was selected at the midpoint of the bone tunnel for 3D imaging. The resulting 3D images were analyzed quantitatively for bone volume fraction (BV/TV), trabecular separation (Tb.SP), trabecular thickness (Tb.Th), and trabecular number (Tb.N). The animal study was approved by the Institutional Animal Care and Use Committee (IACUC) at Chongqing Medical University.

### 2.8 Statistical analysis

Data analysis was performed using GraphPad Prism software (GraphPad Software Inc., USA). Results are presented as the mean ± standard deviation (SD). Independent-samples t-tests were used to compare two groups, while analysis of variance (ANOVA) was employed to evaluate differences among multiple groups.

## 3 Results

### 3.1 Characteristics of SIM-loaded MBG bone substitutes

The morphology of the calcined MBG was examined by TEM and SEM. Fig. 2B and C shows the specific morphology of the MBG nanoparticles according to their size. The MBG sample exhibited a relatively loose surface with an overall porous structure, and large MBG particles were formed by nanoparticle accumulation [31]. The mesopores were identified using the nitrogen adsorption–desorption method to further understand the structure and organization of MBG. Fig. 2D shows an X-shaped curve and H4 hysteresis loops at relative pressures (P/P0) between 0.2 and 0.8, consistent with those of mesoporous MBG particles with pores sized 1–2 nm (Fig. S1). Almost no large pores were observed, indicating that the MGB sample had highly consistent pore structures with narrow and uniformly distributed pores. This distribution characteristic endows MBG with a high specific surface area (Table S2), which provides abundant active sites and superior drug-loading capacity when it is used as a bioactive material [32].

Fig. 2E shows the FTIR spectra of MBG soaked in SBF for 7 and 14 d. Characteristic MBG peaks are observed at 455, 800, and 1080 cm−1, confirming that the glass framework of the sample does not change. In addition, mineralized MBG showed new double-peak absorption bands at 607 and 556 cm−1 that correspond to P–O bending vibrations, indicative of the formation of hydroxyapatite. Fig. 2F displays the XRD patterns of the MBG after it was soaked in SBF for 7 and 14 d. The XRD patterns reveal characteristic diffraction peaks of hydroxyapatite crystals at 2θ = 31.42° and 15.2°, which is consistent with the FTIR data and further confirms that MBG rapidly forms hydroxyapatite. Fig. 2G shows the FTIR spectra of SIM-loaded MBG, which reveals drug-specific C=O and -CH2 absorption bands. Furthermore, the XPS profile of SIM/MBG showed a drug-specific O–C=O band, confirming the successful loading of the drug onto MBG (Fig. 2H).

### 3.2 Characterizing the PTM scaffolds

The molecular weights of the copolymers were determined by GPC, and the results are listed in Table S3. PLTMC has a high molecular weight and a broad molecular weight distribution. The number-average molecular weight (Mn) was 396,970 g/mol, whereas the weight-average molecular weight (Mw) was 945,940 g/mol. These values indicate the presence of a significant number of high-molecular-weight components in the sample. PLTMC has a polydispersity index (PDI) of 2.383, which is indicative of a sample with a wide molecular weight distribution. These characteristics potentially endow PLTMC with good mechanical properties and biocompatibility for bone repair applications.

**Fig. 3:**
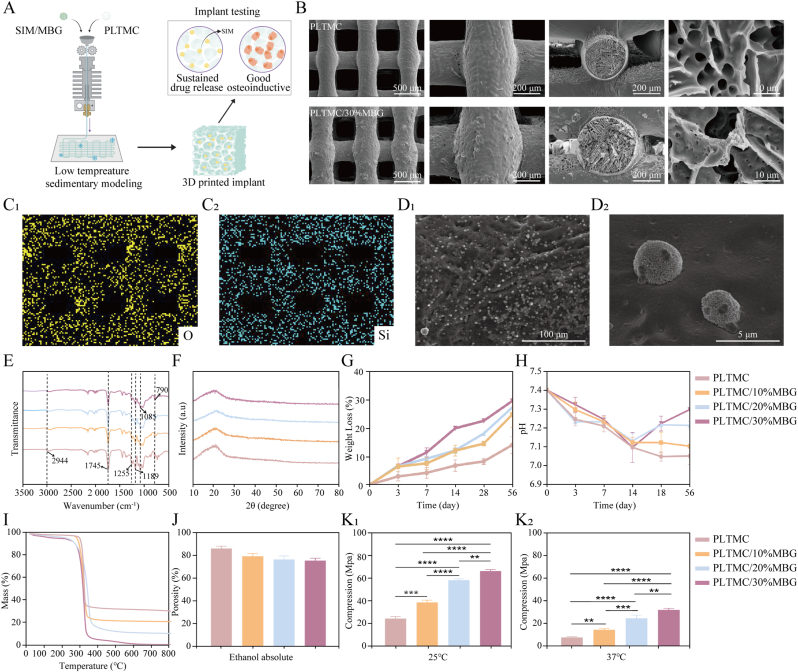
Morphological characteristics and comprehensive performance of PTM scaffolds. (A) 3D low-temperature printing flowchart for the PTM scaffolds. (B) SEM images of the scaffolds. (C) EDS elemental diagrams of the surface of the scaffolds. (D) SEM images of the scaffolds after mineralization. (E) FTIR and (F) XRD of the scaffolds. (G) Weight loss and (H) pH changes of the scaffolds at 37 °C. (I) Thermogravimetric and (J) porosity of the scaffolds. (K) Mechanical properties of the stent at 25 °C and 37 °C. Data are presented as the mean ± SD (*n* = 3); statistical significance was determined by one-way ANOVA with Tukey's post hoc test.

Fig. 3A shows that porous PTM scaffolds containing various proportions of MBG were successfully printed using low-temperature 3D printing technology. Scaffolds containing 0 % MBG (PLTMC/0 %MBG), 10 % MBG (PLTMC/10 %MBG), 20 % MBG (PLTMC/20 %MBG), and 30 % MBG (PLTMC/30 %MBG) were prepared based on the graded proportions of MBG in the mixture. The surface morphology of the 3D-printed stent changed significantly with increasing MBG content; the printed slurry could not be well shaped when the MBG content exceeded 30 %. The specific printing conditions are shown in Fig. S2. While the PLTMC stent exhibited a smooth surface, it became increasingly rough with increasing MBG content. The PLTMC/10 %MBG and PLTMC/20 %MBG scaffolds had an excessive number of particles with irregular structures on their surfaces (Fig. S3). The PLTMC/30 %MBG scaffold exhibited the roughest surface area. These observations revealed that the addition of MBG significantly affected the surface morphology and microstructure of the scaffold, thereby increasing surface roughness and complexity. Meanwhile, cross-sectional SEM revealed 2–10 μm pores within the scaffold (Fig. 3B), which is the critical pore size range for osteogenesis [33]. These pores facilitate nutrient diffusion (e.g., oxygen and glucose) and metabolic waste removal, ensuring cell viability in deeper scaffold regions. Furthermore, interconnected micropores act as conduits for growth factors, supporting osteoprogenitor recruitment and mineralization. Hybrid scaffolds integrating 2–10 μm micropores with macropores (>100 μm) synergize hierarchical bone regeneration; specifically, micropores optimize early cell–matrix interactions, whereas macropores later enable vascularization and osteoclast-mediated remodeling [34].

Fig. 3C and D shows the EDS and SEM images, respectively, of the scaffolds with PLTMC/30 %MBG immersed in simulated body fluids for 7 d. EDS revealed that MBG was uniformly distributed on the surface of the stent (Fig. 3C). The SEM analysis indicated that as the MBG content increased, the degree of mineralization in the PTM scaffolds increased considerably. The PLTMC samples exhibited almost no mineralization, whereas an increasing number of mineralized sediments gradually appeared on the surfaces of the PLTMC/10 %MBG, PLTMC/20 %MBG, and PLTMC/30 %MBG samples (Fig. S4). The surface mineralized particles on the PLTMC/30 %MBG sample were the densest and most discernible, suggesting the highest degree of mineralization (Fig. 3D). These results indicate that introducing MBG significantly promotes the mineralization of the composite materials, which helps enhance their biological activity and bone integration ability in bone repair applications.

Fig. 3E shows the FTIR spectra of the PTM stents. Each composite scaffold exhibited a clear absorption peak at 2944 cm−1, which is attributable to C–H stretching vibrations associated with the LA–CH and -CH3 moieties, as well as the TMC–CH2 units in the polymer [35]. Meanwhile, the composite scaffolds exhibit similar absorption peaks at 1744, 1255, and 1189 cm−1 that are attributable to the C=O and C–O–C stretching vibrations associated with the TMC and LA segments, consistent with the presence of PLTMC in the composite scaffold. The absorption peaks at 1085, 790, and 466 cm−1 are attributable to asymmetric Si–O–Si stretching, symmetric Si–O stretching, and symmetric Si–O–Si bending, respectively, consistent with the presence of MBG in the composite scaffold. Characteristic peaks associated with the PLTMC and MBG composite materials were still clearly visible after blending, printing, and freeze-drying. Thus, it was confirmed that MBG had been successfully dispersed in the composite material in a manner that changed the microstructure of the material and altered its molecular environment without damaging its main structure [36].

Fig. 3F shows the XRD pattern of the PTM composite. Each component of each PTM scaffold shows peaks with specific shapes. In particular, the peak at 2θ = 22° reveals that the scaffold is essentially amorphous [37]. Fig. 3G shows that the rate of mass loss of the stent increased with increasing degradation time, and faster degradation was observed with increasing MBG content. The highest stent mass-loss rate of 28.4 % was observed in the eighth week for PLTMC/30 %MBG. These results indicate that internal MBG was released during the degradation process owing to the degradation of the stent surface, which consequently affected the rate of mass loss. Fig. 3H shows the changes in pH during stent degradation. All the degradation solutions had initial pH values of 7.4. The initial pH reduction was attributed to acidic degradation products (e.g., LA) released through ester bond cleavage during PTM scaffold hydrolysis. Notably, the system maintained pH homeostasis within the physiologically neutral range (approximately 7.0) throughout the degradation process. The addition of MBG promotes the acidic degradation of the scaffold because the silicates and calcium salts in MBG gradually dissolve during degradation, releasing the silicate and calcium ions that form silicates in water. Subsequent reactions with water generate hydroxide ions (OH−) that increase the pH of the solution such that it becomes alkaline [38].

Thermogravimetric analysis traces for the various PTM materials are shown in Fig. 3I, which reveals that PLTMC begins to degrade at approximately 200 °C. This result indicates that the material is thermally stable at this temperature. Degradation occurs rapidly at around 340 °C, leaving almost no residue. PLTMC/10 %MBG begins to degrade at a temperature similar to that of PLTMC; degradation proceeds rapidly at around 360 °C, leaving a residual mass of about 10 %. Conversely, PLTMC/20 %MBG begins to degrade at a slightly higher temperature (∼275 °C), and rapid degradation occurs at around 350 °C, leaving a residual mass of about 20 %. PLTMC/30 %MBG exhibited the highest initial degradation temperature of approximately 300 °C, with rapid degradation observed at around 350 °C, and the residual mass was ∼30 %. Overall, these results revealed that increasing the proportion of MBG significantly improved the thermal stability and structural retention of the PLTMC composite. The temperature at which the composite material initially decomposed increased with increasing MBG content, and larger amounts of residual mass were obtained at high temperatures. Hence, the MBG reinforced the composite material and increased its thermal decomposition temperature and residual mass, thereby improving its thermal stability.

The porosity of the scaffolds was determined using the water displacement method. Fig. 3J shows that the porosity of the pure PLTMC scaffold was 85.17 %. Upon the addition of 10 %, 20 %, and 30 % MBG, the porosities decreased to 78.5 %, 75.72 %, and 74.76 %, respectively. In summary, the prepared scaffolds met the porosity requirement of 50 %–90 % for cancellous bone [39]. Fig. 3K illustrates the mechanical performance of the PTM stents. It can be observed that the compressive modulus correlates positively with the MBG content because MGB uniformly adheres to the stent, thereby enhancing its ability to resist deformation. Fig. 3K1 shows the compressive moduli of the various stents at 25 °C. The scaffold of the PLTMC component exhibited an average compression modulus of 24.53 MPa, whereas that of the PLTMC/30 %MBG component exhibited a high value of 66.33 MPa. Fig. 3K2 shows the compressive moduli of the stents at 37 °C. The average compression modulus of the PLTMC stent was 7.89 MPa, whereas that of the PLTMC/30 %MBG stent was 32.10 MPa. These results reveal that the compression moduli of the MBG scaffolds meet the requirements of cancellous bone regardless of whether the temperature is 25 or 37 °C. Accordingly, PLTMC/30 %MBG, which demonstrated the best comprehensive performance in the SIM drug release experiments was selected. According to the literature, 0.01–1 μM SIM promotes osteogenic differentiation, while concentrations above 1 μM significantly inhibit cell proliferation. PTM/200 SIM released more than 1 μM of the drug in the first week, while PTM/20 SIM and PTM/100 SIM exhibited sustained release at concentrations in the 0.01–1 μM range (Fig. S5). Therefore, the low-concentration 20 SIM/PTM and high-concentration 100 SIM/PTM scaffolds were selected for subsequent biological experiments.

### 3.3 Shape-memory effect of the PTM composite scaffold

**Fig. 4:**
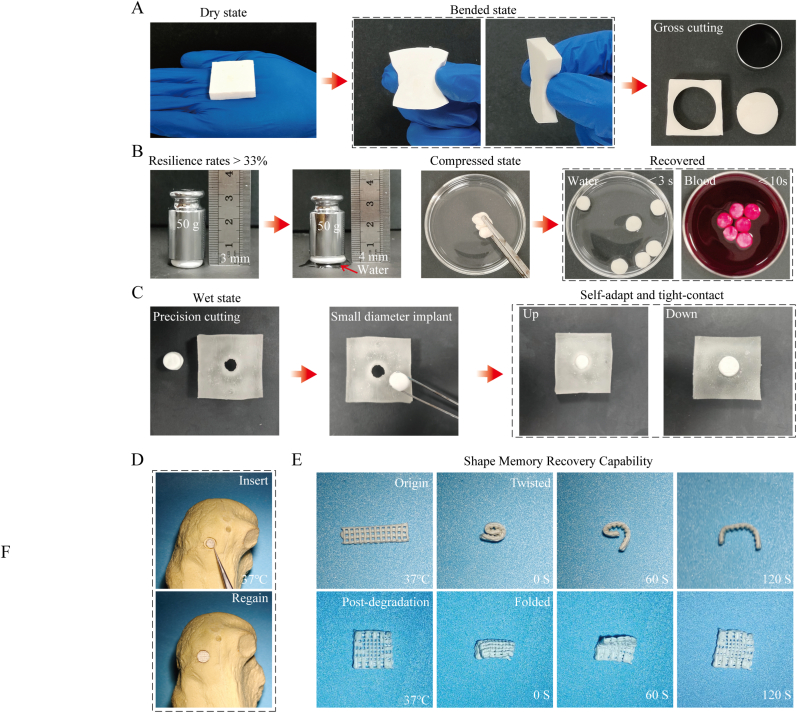
Operability and shape-memory effect of PTM scaffolds. (A) The PTM scaffolds can be arbitrarily trimmed at 37 °C to achieve implantation in different bone defects, (B) The PTM scaffolds returns to its original shape after being compressed with a 100 g weight in water and blood at 37 °C. (C) The PTM scaffolds closely follow the defect contour after shape-memory activation. (D) The PTM scaffold completely filling the defect area. (E) The shape memory function of the PTM scaffold and the post-degradation scaffold.

The processability and shape-memory function of a bone implant during surgery are crucial for the personalized customization of irregular bone defects [40]. The DSC results shown in Fig. S3 reveal that PTM has a Tg of 37.90 °C, which is similar to that of the human body. Hence, this material exhibits good elasticity and shaping ability at body temperature (Fig. S6). Fig. 4A shows that the PTM composite stent could be trimmed as required during surgery. The shape of the trimmed implant recovered within 10 s in water and blood at 37 °C (Fig. 4B). The operability and shape-memory capabilities of the composite stent enabled it to swiftly revert to its original configuration and conform to the contours of the bone defect, thereby ensuring seamless integration, as shown in Fig. 4C. Consequently, it was established that the PTM composite scaffold is a shape-memory-effect material with an exceptionally rapid shape recovery rate and short recovery time. In vitro simulation demonstrated that the PTM scaffold effectively filled the bone defect area, providing structural support and conforming to irregular anatomical contours. To evaluate the functional sustainability of the material, its shape-memory capability was systematically investigated for both pristine PTM scaffolds and their degraded counterparts under simulated physiological conditions (Fig. 4D). After controlled thermal cycling tests, the original scaffold mostly recovered its shape within 120 s at 37 °C, whereas the degraded scaffold completely recovered its shape within 120 s, even after 12 weeks of degradation (Fig. 4E). These findings highlight the potential of PTM scaffolds as dynamic implants capable of adapting to defect morphology and sustaining biomechanical integrity during tissue remodeling.

### 3.4 Detecting induction and SIM/PTM scaffold biocompatibility

**Fig. 5:**
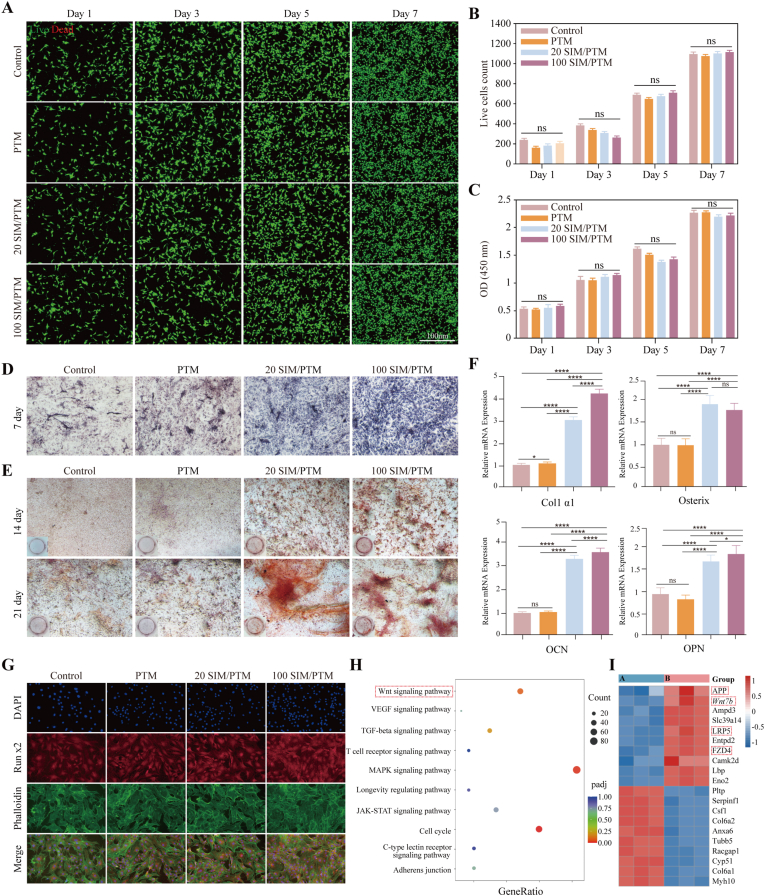
In vitro biocompatibility and osteogenic induction ability of the PTM scaffolds. (A) Control group (without PTM scaffolds) and the different PTM scaffolds extract groups were tested with live/dead fluorescence stain assays. (B) Comparative analysis of the number of viable cells between the control group and each PTM scaffold group. (C) Assessment of MSC viability using the CCK-8 assay within the extracts of PTM scaffolds. (D) ALP staining was performed in BMSCs at 7 days. (E) ARS staining was performed in BMSCs at 14 and 21 days. (F) qPCR evaluation of the mRNA expression of Col1 α1, Osterix, OCN, and OPN in BMSCs treated with different PTM scaffold extracts for 7 days. (G) Immunofluorescence staining in BMSCs treated with different PTM scaffold extracts for 7 days. (H) KEGG analysis of RNA sequences. (I) Heat map of migrating genes (A: Control; B: 100 SIM/PTM) (∗P < 0.05, ∗∗P < 0.01, ∗∗∗P < 0.001, ∗∗∗∗P < 0.0001, n = 3).

The biocompatibility of the SIM/PTM scaffolds was initially evaluated through in vitro culture using scaffold extracts. Fig. 5A shows the live/dead assay results, where green and red fluorescence indicate viable and nonviable cells, respectively. The majority of cells remained viable, with only a few dead cells observed. Over time, viable cell proliferation was substantial, with no significant differences between the groups (Fig. 5B). The CCK8 assay results further supported the findings from the live/dead staining (Fig. 5C). Collectively, these experimental data suggested that the SIM/PTM scaffold was not significantly cytotoxic and was suitable for use in subsequent experiments.

ALP and ARS were used to examine the ability of the composite scaffold extract to promote osteogenesis. Fig. 5D and E shows that the 100 SIM/PTM group exhibited the darkest color, the most calcium nodules, and the best osteogenicity; the quantitative results are presented in Fig. S8. RT-PCR was used to detect the expression of osteogenic marker genes (col1 a1, OCN, OSX, and OPN) on the seventh day. The results revealed that the four osteogenesis-related genes in the 20 SIM/PTM and 100 SIM/PTM composite scaffolds were expressed significantly more than those in the control and PTM groups. In particular, col1 a1, OCN, and OPN were expressed more in the 100 SIM/PTM group than in the 20 SIM/PTM group (Fig. 5F). Immunofluorescence analysis showed that Runx2 and BMP2 were expressed significantly more in MSCs of the 20 SIM/PTM and 100 SIM/PTM groups than in those of the control and PTM groups (Fig. 5G and S8). We also tested the pro-angiogenic activity of the stent. The scratch test and RT-PCR results showed that the 20SIM/PTM and 100SIM/PTM scaffolds effectively promoted the migration and angiogenic ability of vascular endothelial cells (Fig. S9). The potential mechanism responsible for the ability of the scaffold to promote osteogenic differentiation in bone marrow MSCs was examined using RNA sequencing (RNA-seq) in the control and 100 SIM/PTM groups (with the best osteogenic effect) to analyze gene expression during bone regeneration. Differential gene expression analysis identified notable changes in the BMSCs of the 100 SIM/PTM group compared to the control group, with 1331 genes upregulated and 3004 genes downregulated, as shown in Fig. S10. Furthermore, KEGG enrichment analysis indicated the activation of several osteogenesis-related pathways in the 100 SIM/PTM group, including the Wnt signaling pathway (Fig. 5H). In particular, in the BMSCs, 100 SIM/PTM appeared to regulate the expression of App, Wnt 7b, Lrp5, and Fzd4 among the genes upregulated in the Wnt signaling pathway; these differentiation-related genes have been shown to be associated with osteogenesis (Fig. 5I).

### 3.5 In vivo bone defect repair by the SIM/PTM scaffolds

**Fig. 6:**
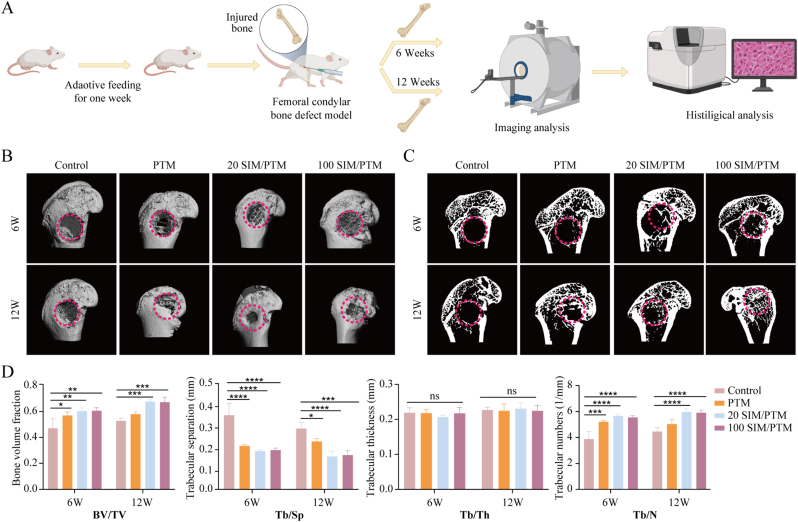
Implantation of PTM in vivo induces repair of bone defect areas. (A) Schematic diagram of animal modeling and experiment. (B) Micro CT 3D reconstruction images of defect sites at different time points after stent implantation. (C) Micro CT cross-sectional image of the defect site. (D) Newly formed bone mass (BV/TV, BV newly formed bone volume, TV total volume), trabecular separation (Tb. Sp), thickness of newly formed bone trabeculae (Tb. Th), and number of newly formed bone trabeculae (Tb. N) (∗P < 0.05, ∗∗P < 0.01, ∗∗∗P < 0.001, ∗∗∗∗P < 0.0001, n = 5).

We further investigated the osteogenic properties of SIM/PTM scaffolds in a rat femoral defect model (Fig. 6A). The 3D reconstruction of the micro-CT scan displayed in Fig. 6B shows that the PTM scaffold was tightly integrated with the natural bone tissue and matched its shape. The cross-sectional image showed new bone growth in the composite scaffold (Fig. 6C). A quantitative assessment of BV/TV, TB.Sp, Tb.Th, and Tb.N revealed that the 20 SIM/PTM and 100 SIM/PTM groups exhibited extensive, low-dispersion, new bone formation within the scaffolds (Fig. 6D).

**Fig. 7:**
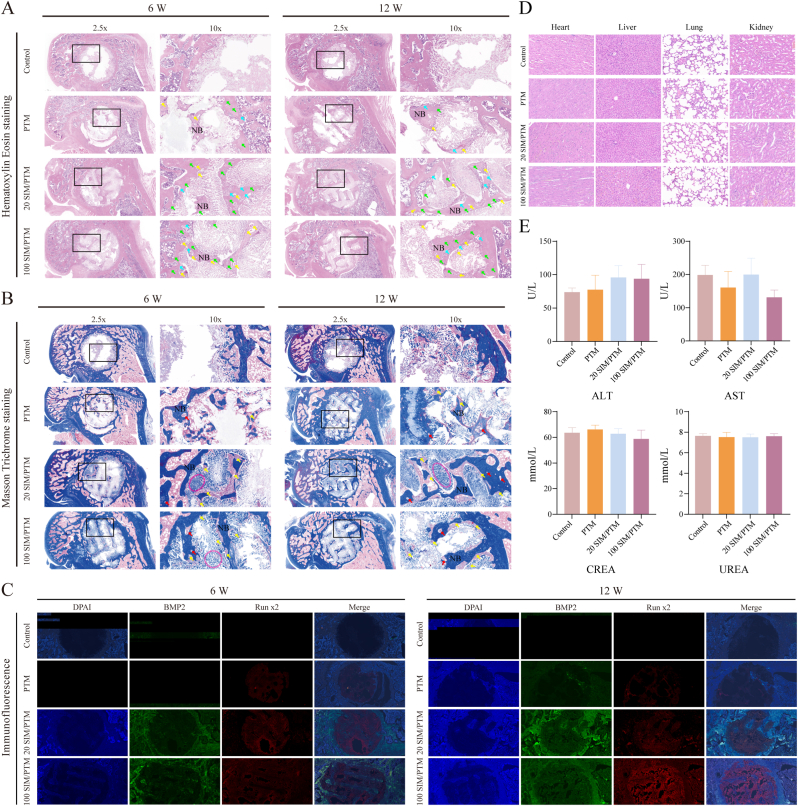
Evaluation of new bone formation in bone defect areas at 6 and 12 weeks after scaffold implantation. (A) H&E staining and (B) Masson staining of the bone defects in different groups. NB:new bone; green arrow: osteocyte; blue arrow: osteoblasts; yellow arrow: blood vessel; red arrow: vascular system; pink dashed area: fibrous callus. (C) Immunofluorescence images of the tissue osteogenic marker BMP2 and Runx2 in vivo. (D) H&E staining of the heart, liver, lung, and kidney of rats 12 weeks after operation. (E) Liver and kidney functions of rats in each group. (For interpretation of the references to color in this figure legend, the reader is referred to the Web version of this article.)

The bone-repair process was further analyzed using H&E and Masson's trichrome staining (Fig. S11). No inflammatory cells were observed in any of the groups following stent implantation. Large fat bubbles, numerous blood vessels (yellow arrows), osteoblasts (blue arrows), and bone cells (green arrows) accumulated in the bone defect area in the control and PTM groups, particularly in the 20 SIM/PTM and 100 SIM/PTM groups (Fig. 7A). Fig. 7B shows significant new bone growth in the bone defect area of the stent group. A large number of vascular systems are visible within the new bone (red arrows), and bone regeneration is visible along the stent direction. Large amounts of fibrous calli tightly connected to the new bone (red dashed area) were observed in the stents of the 20 SIM/PTM and 100 SIM/PTM groups after 6 and 12 weeks of stent implantation, highlighting the bone regeneration ability of the composite stent. Immunofluorescence analysis was used to determine the expression of BMP2 and Runx2, which are osteogenic proteins, in the newly formed bone on the scaffold in the defect area. The 100 SIM/PTM group exhibited higher expressions of BMP2 and Runx2 than the other groups (Fig. 7C), which was consistent with the in vitro cell experiments.

In summary, SIM/PTM scaffolds promoted bone regeneration in bone defect areas. Heart, liver, lung, and kidney tissue sections were prepared, and morphological changes were observed following H&E staining. No abnormalities or pathological changes were observed in the control and experimental groups (Fig. 7D). The experimental group exhibited normal levels of aspartate aminotransferase (AST), alanine aminotransferase (ALT), creatinine (CREA), and urea nitrogen (UREA), with no significant differences compared to the control group (Fig. 7E). These results demonstrated that the composite scaffold was highly biocompatible in vivo.

## 4 Discussion

Bone defect repair using conventional shape-memory materials faces critical challenges because of their mechanical and biological incompatibility, as these materials often exhibit inadequate biodegradability and suboptimal integration with natural bone tissue [[41], [42], [43], [44], [45], [46]]. To address these limitations, we engineered a PTM composite scaffold via 3D printing, optimizing shape-memory functionality and bioactivity. By tuning the LA/TMC monomer ratio, the PLTMC matrix achieves a glass transition temperature (*T*g) of 25–37 °C, enabling intraoperative shape adaptation; specifically, compressed scaffolds rapidly regain their predefined porous architectures at body temperature, conforming seamlessly to defect geometries while minimizing surgical trauma [47]. PLTMC exhibits a tunable glass transition temperature (*T*g: ∼35–40 °C) aligned with physiological conditions, enabling body-heat-triggered shape recovery for minimally invasive applications. In addition, it avoids the impractically low *T*g of PCL (−60 °C) and the high *T*g of PLGA (45–55 °C), which requires external heating. This innovation ensures safe, precise deployment and degradation-synced mechanical stability, positioning PLTMC as an ideal scaffold material for dynamic biomedical devices. Incorporating 30 % MBG enhanced the compressive strength to the scaffold and roughened its surface, promoting osteoblast adhesion and migration [48]. The mesoporous structure of MBG facilitated sustained ion exchange (Ca^2+^, PO_4_^3−^) in physiological environments, catalyzing biomimetic mineralization that improved scaffold–bone integration and osteogenic differentiation. Crucially, MBG integration mitigated acidic degradation byproducts typical of polylactic acid, while the surface-eroding degradation profile of the composite preserved mechanical integrity during bone regeneration [49]. The hierarchical porosity of the scaffold, achieved through low-temperature rapid prototyping, mimicked the trabecular structure of natural bone, balancing its load-bearing capacity (66.33 ± 1.44 MPa) with nutrient diffusion. However, excessive MBG (>40 %) compromised printability, whereas the 30 % MBG formulation showed the optimal balance of bioactivity, mechanical stability, and structural fidelity. This dual-functional design overcomes key limitations of traditional shape-memory alloys (e.g., the non-degradability of Ni–Ti [43]) by synergizing stimuli-responsive shape adaptation with bioactive mineralization, enabling personalized repair of complex defects. The dynamic compliance of the scaffold with host tissue mechanics and its ability to spatiotemporally control mineral deposition positions it as a transformative solution for high-load bone regeneration, bridging the gap between synthetic material performance and biological repair mechanisms.

The localized delivery of osteoinductive agents such as SIM in BTE requires precise control over drug release kinetics to balance therapeutic efficacy and adverse effects [50]. While SIM enhances bone repair by activating osteoblast differentiation and angiogenesis, systemic administration suffers from hepatic metabolism, and high local concentrations risk inflammatory responses or impaired healing [[51], [52], [53]]. To address this, we engineered a mesoporous bioactive PTM composite scaffold via low-temperature 3D printing, preserving SIM bioactivity while creating hierarchical pore structures [[54], [55], [56]]. The mesopores of MBG enabled high SIM loading, while the surface erosion of PLTMC ensured a gradual drug release profile aligned with the timeline of bone regeneration [57]. Unlike bulk degradation in conventional polymers, surface erosion progressively exposes SIM through microscale material regression, maintaining therapeutic concentrations (optimized at 0.1–1 μM) that avoid endothelial cytotoxicity while promoting angiogenesis—a critical balance given SIM's biphasic effects on vascular endothelial cells (e.g., pro-angiogenic at low doses vs. anti-proliferative at >5 μM) [58]. This degradation-profile synergy with the scaffold architecture—i.e., secondary pores that mimic the trabecular network of natural bone—enhanced osteoblast adhesion and nutrient exchange while providing sustained mechanical support during defect remodeling [59,60]. The compressive strength of the scaffold (66.33 ± 1.44 MPa at 30 % MBG) and its gradual strength transfer prevented stress shielding, which is critical for high-load repair. Crucially, the ion-exchange capacity of MBG amplified the osteogenic effects of SIM by enriching the microenvironment with Ca^2+^/PO_4_^3−^, fostering biomimetic mineralization and scaffold–host integration [49]. By harmonizing the material degradation kinetics with drug release profiles, this system overcomes the limitations of rapid-release carriers (e.g., burst-induced toxicity) and non-degradable implants (e.g., persistent foreign-body responses), achieving spatiotemporal control over osteoinduction. The dual functionality of the structural support and bioactive delivery positions the scaffold as a new paradigm in defect repair, where mechanical stability and biochemical signaling evolve synergistically with tissue regeneration [61].

Several studies have demonstrated that SIM enhances osteogenesis by activating the Wnt/β-catenin signaling pathway, which plays a crucial role in osteoblast differentiation and bone formation [62]. This pathway is typically activated through the inhibition of glycogen synthase kinase-3β (GSK-3β), a major suppressor that facilitates the degradation of β-catenin. By inhibiting GSK-3β activity, SIM prevents the degradation of β-catenin, leading to its accumulation and translocation into the nucleus [63]. Through the activation of the Wnt/β-catenin pathway, SIM enhances the synthesis of bone matrix proteins, such as type I collagen (Col-I) and osteocalcin, which are essential for bone mineralization and tissue formation, thereby promoting the deposition and mineralization of new bone tissue [64]. Furthermore, SIM activates the BMP2 signaling pathway, which further enhances osteogenesis. SIM's dual activation of the BMP2 and Wnt/β-catenin pathways creates a synergistic osteogenic loop. The BMP2 pathway upregulates Smad1/5/8 phosphorylation, which directly interacts with β-catenin (stabilized by SIM-mediated GSK-3β inhibition) to form transcriptional complexes at promoters of osteogenic genes such as Runx2 and osterix [65]. This Smad–β-catenin crosstalk amplifies the expression of bone matrix proteins (e.g., Col-I and osteocalcin) and mineralization regulators (e.g., ALP) [66]. Overall, the dual activation by SIM of the Wnt/β-catenin and BMP2 pathways contributes to osteogenesis, offering promising potential applications in bone defect repair and osteoporosis treatment. These results suggest that SIM may be a novel therapeutic strategy for bone repair and regeneration [67]. Thus, this study builds upon previous research by introducing SIM-loaded MBG with a high surface area and further investigates two concentrations (low and high) based on the drug concentration. ALP and ARS assays showed that the addition of SIM effectively promoted the osteogenic differentiation of mesenchymal stem cells. RT-PCR analysis showed that the 20 SIM/PTM and 100 SIM/PTM groups exhibited significantly higher expression levels than the control and PTM groups. The immunofluorescence results demonstrated that SIM/PTM effectively promoted the expression of BMP2 and Runx2, thereby exerting osteogenic effects. The osteogenic effect was further validated in a rat model of a femoral condyle bone defect. Histological sections showed that with the degradation of the material, the newly formed bone grew in an orderly manner. Notably, the direction of bone ingrowth was consistent with that of the scaffold pores. Within the scaffold, scattered mineralization centers were formed, and a rich myeloid vascular network surrounded the new bone, indicating an abundant blood supply in the newly formed bone and good biological activity. Consequently, the SIM/PTM scaffolds demonstrated excellent osteogenic properties.

## 5 Conclusions

This study demonstrates the successful preparation of high-molecular-weight multifunctional bone repair scaffolds that mimic the macrostructure of cancellous bone using low-temperature 3D printing technology. These scaffolds are highly biocompatible and release SIM in a sustained manner, thereby promoting the osteogenic differentiation of bone marrow mesenchymal stem cells. Bone growth was observed along the direction of the scaffold in a rat femoral condyle bone defect model, which filled the pores within the bone defect area. The developed multifunctional bone scaffold was mechanically supportive, gradually degraded, and promoted osteogenesis, thereby providing a promising strategy for repairing large individualized bone defects.

## CRediT authorship contribution statement

**Xulin Hu:** Writing – review & editing, Writing – original draft, Methodology, Conceptualization. **Shengwen Cheng:** Writing – original draft, Methodology, Conceptualization. **Senrui Liu:** Writing – review & editing, Visualization, Project administration. **Minchang Zhou:** Writing – review & editing, Visualization, Project administration. **Junyan Liu:** Writing – review & editing, Visualization, Project administration. **Jiaying Wei:** Visualization, Data curation. **Yixuan Lan:** Validation, Resources. **Yu Zhai:** Validation, Resources. **Xiaohong Luo:** Visualization, Resources. **Mingfei Dong:** Validation, Resources. **Zu Xiong:** Validation, Resources. **Wei Huang:** Supervision, Project administration. **Chen Zhao:** Supervision, Project administration.

## Declaration of competing interest

The authors declare that they have no known competing financial interests or personal relationships that could have appeared to influence the work reported in this paper.

## Data Availability

Data will be made available on request.
