# Supplementary material for: Fast shape memory function and personalized PLTMC/SIM/MBG composite scaffold for bone regeneration
Source: Mater Today Bio. 2025 May 2;32:101791. doi: 10.1016/j.mtbio.2025.101791 (PMC12098156; doi:10.1016/j.mtbio.2025.101791)
Supplement: Multimedia component 1 [file mmc1.docx]

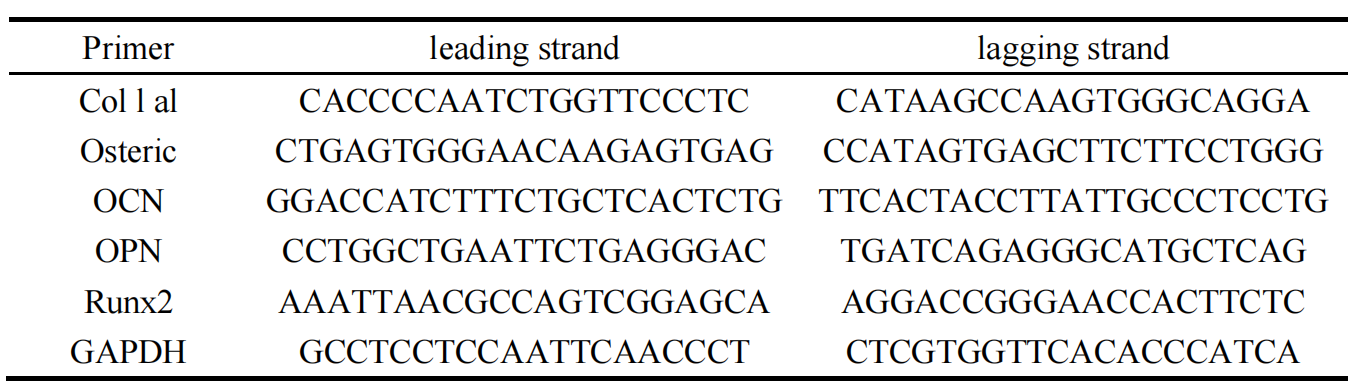


Table S1 RNA primer sequence


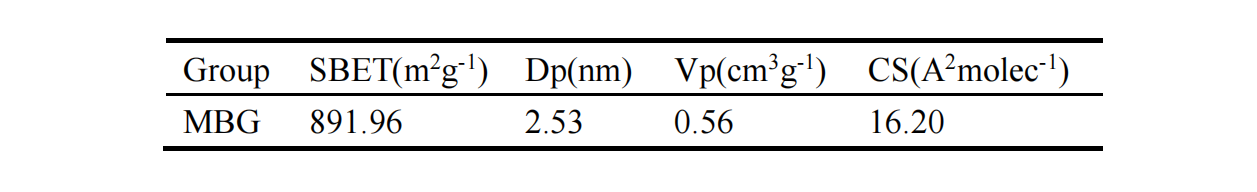


Table S2 Specific surface area of MBG


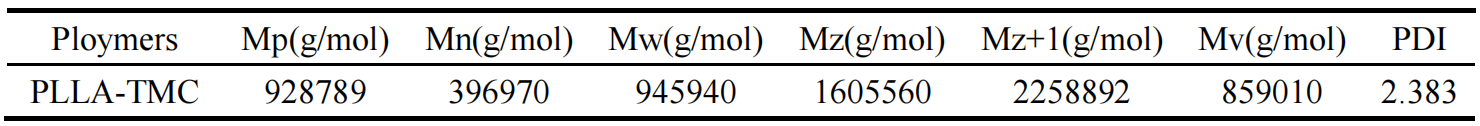


Table S3 Molecular weights of copolymers


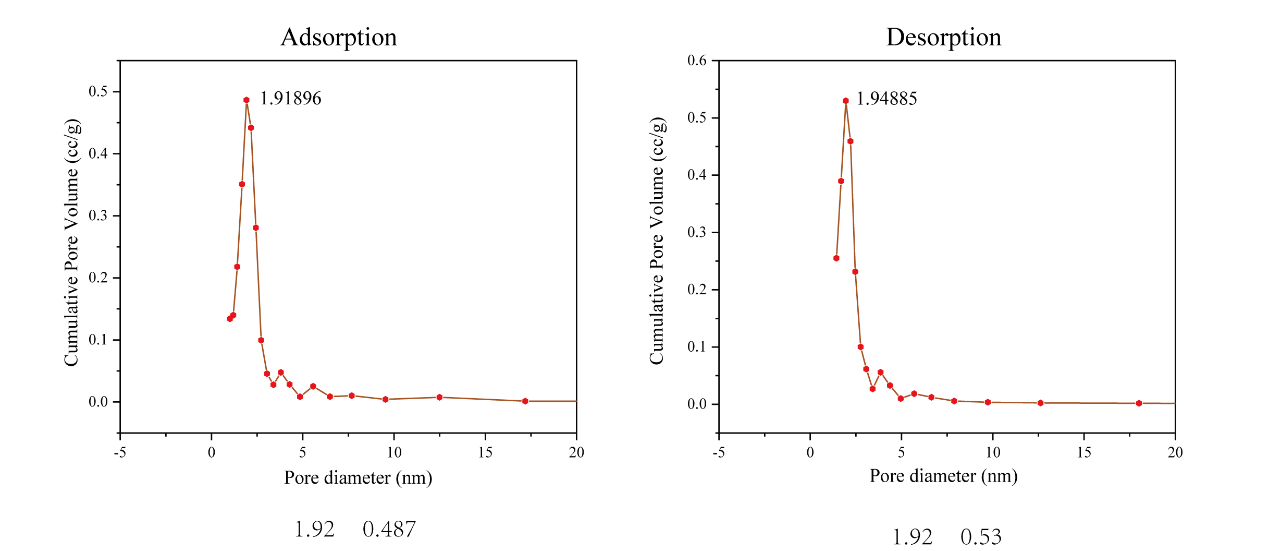


Figure S1 Pore size distribution of MBG


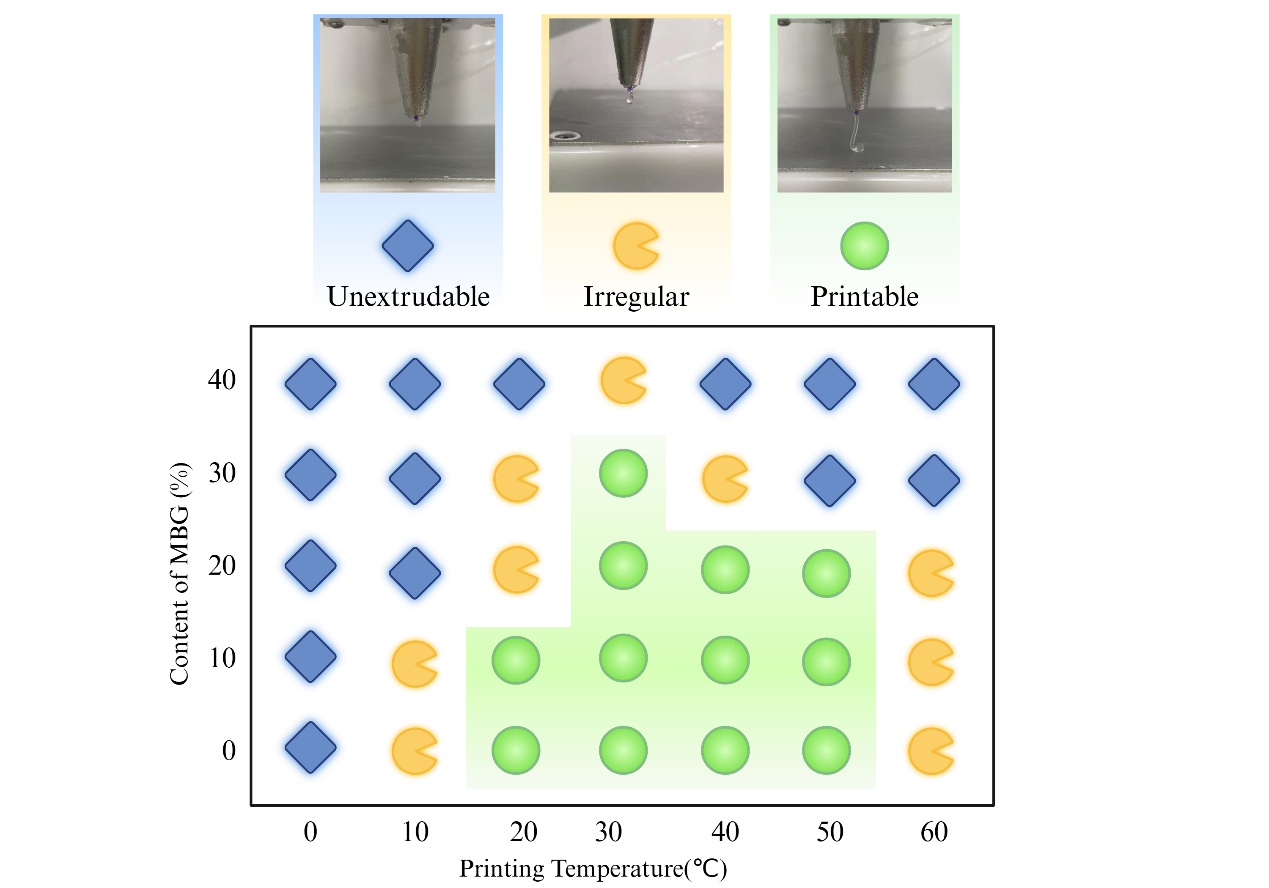


Figure S2 Print condition


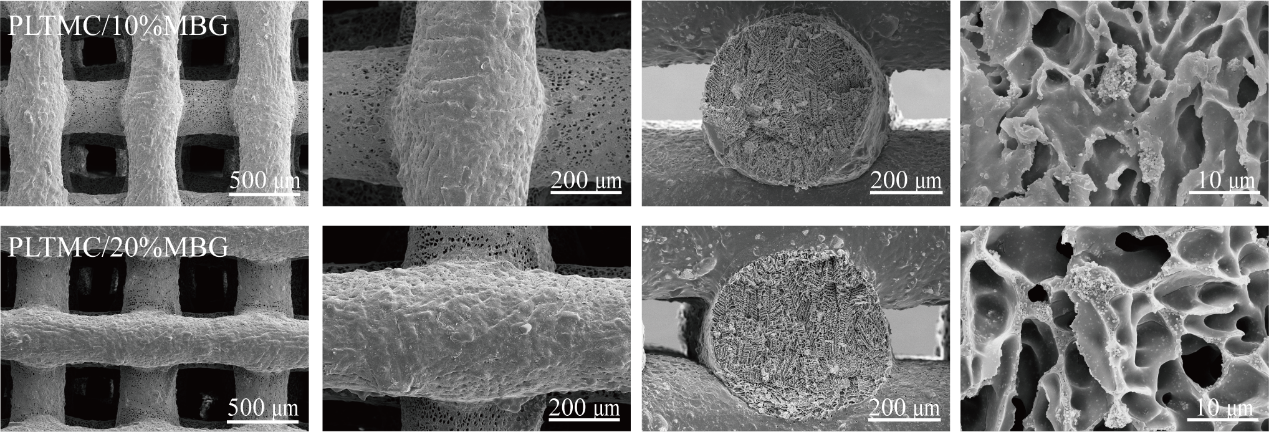


Figure S3 SEM of the scaffold


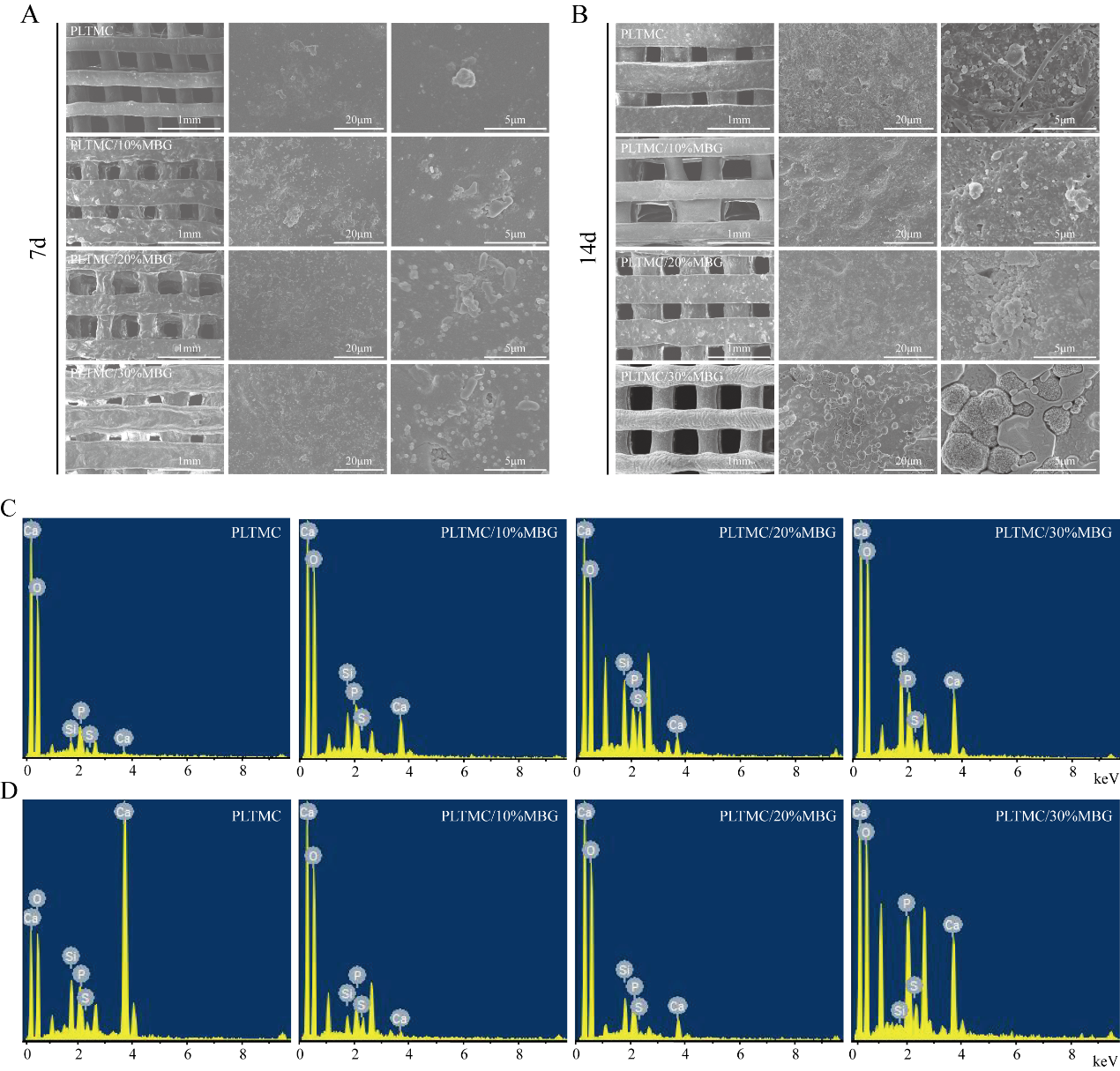


Figure S4 (A)The SEM photos of different scaffolds after immersedin SBF for 7 days. (B)The SEM photos of different scaffolds after immersedin SBF for 14 days. (C)EDS after 7 days of soaking in SBF for different scaffolds. (D)EDS after 14 days of soaking in SBF for different scaffolds.


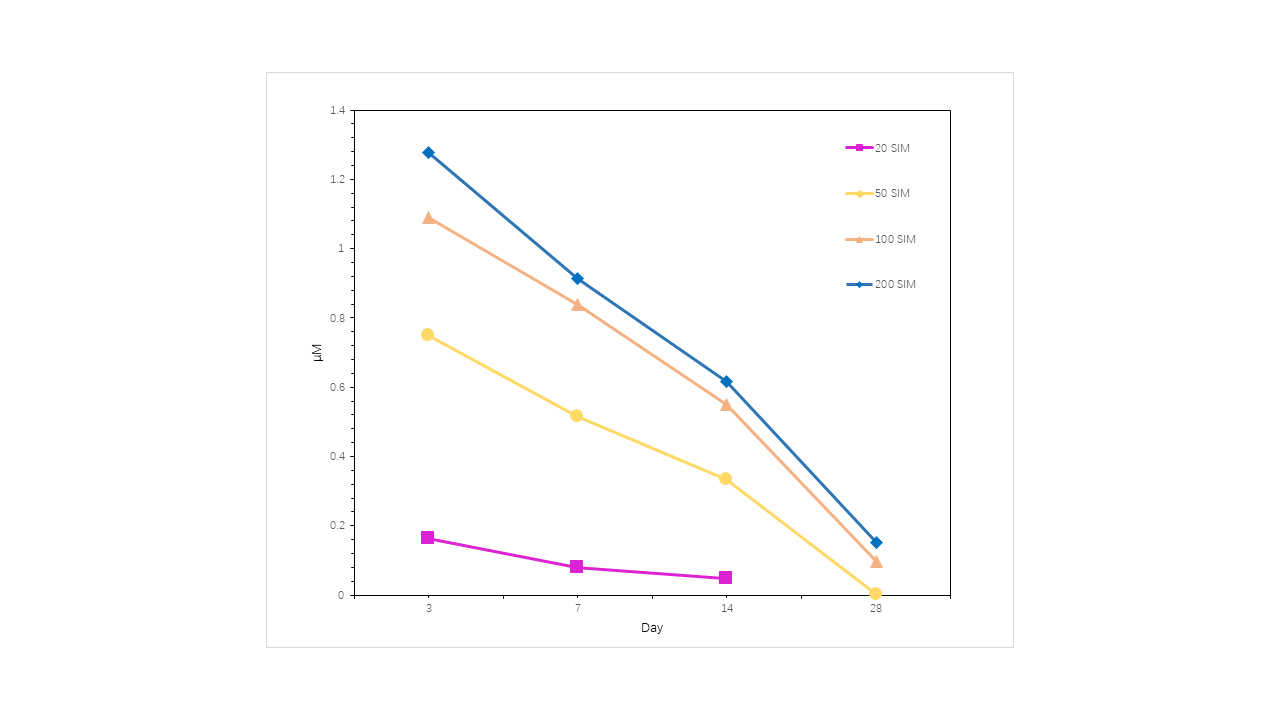


Figure S5 Drug release profiles of 3, 7, 14 and 28 day scaffolds in PBS buffer


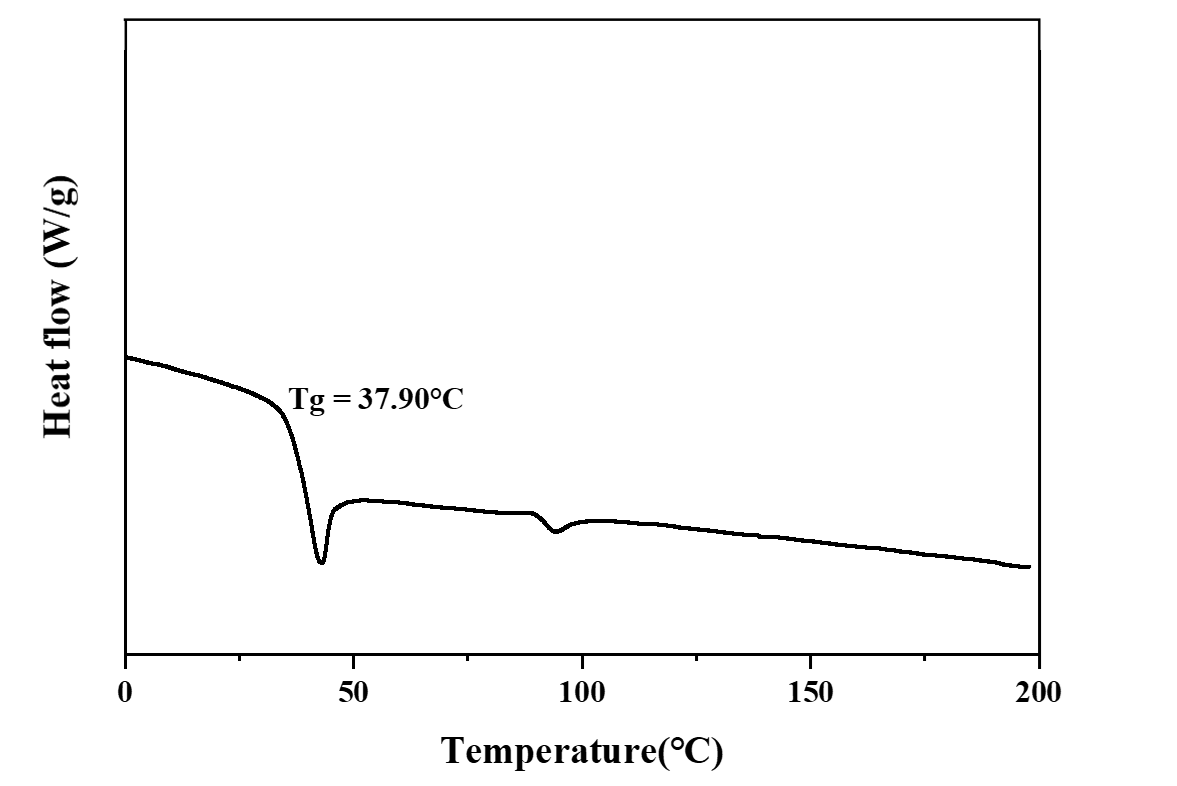


Figure S6 DSC of PLTMC/MBG


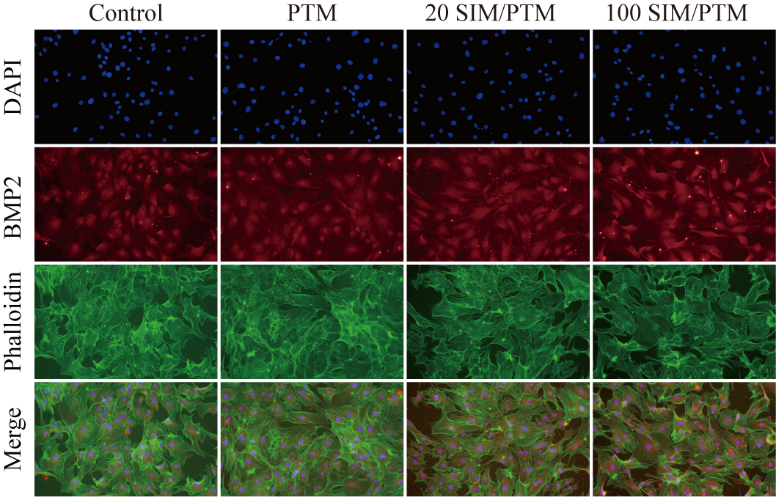


Figure S7 Immunofluorescence staining in BMSCs treated with different PTM scaffolds extract for 7 days.


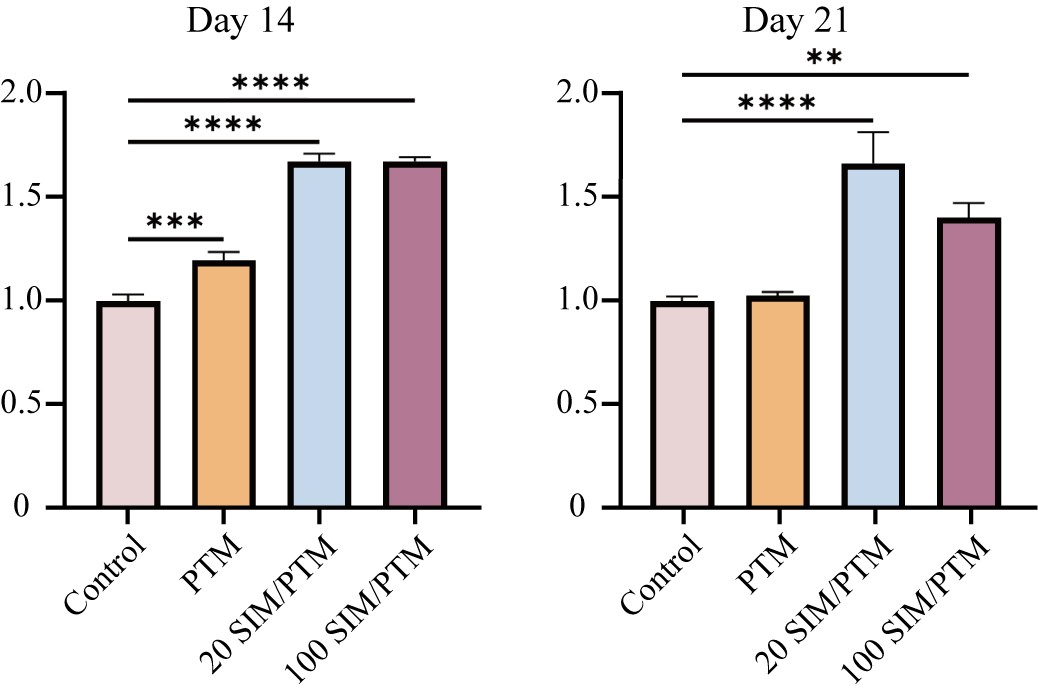


Figure S8 ARS quantitative analysis


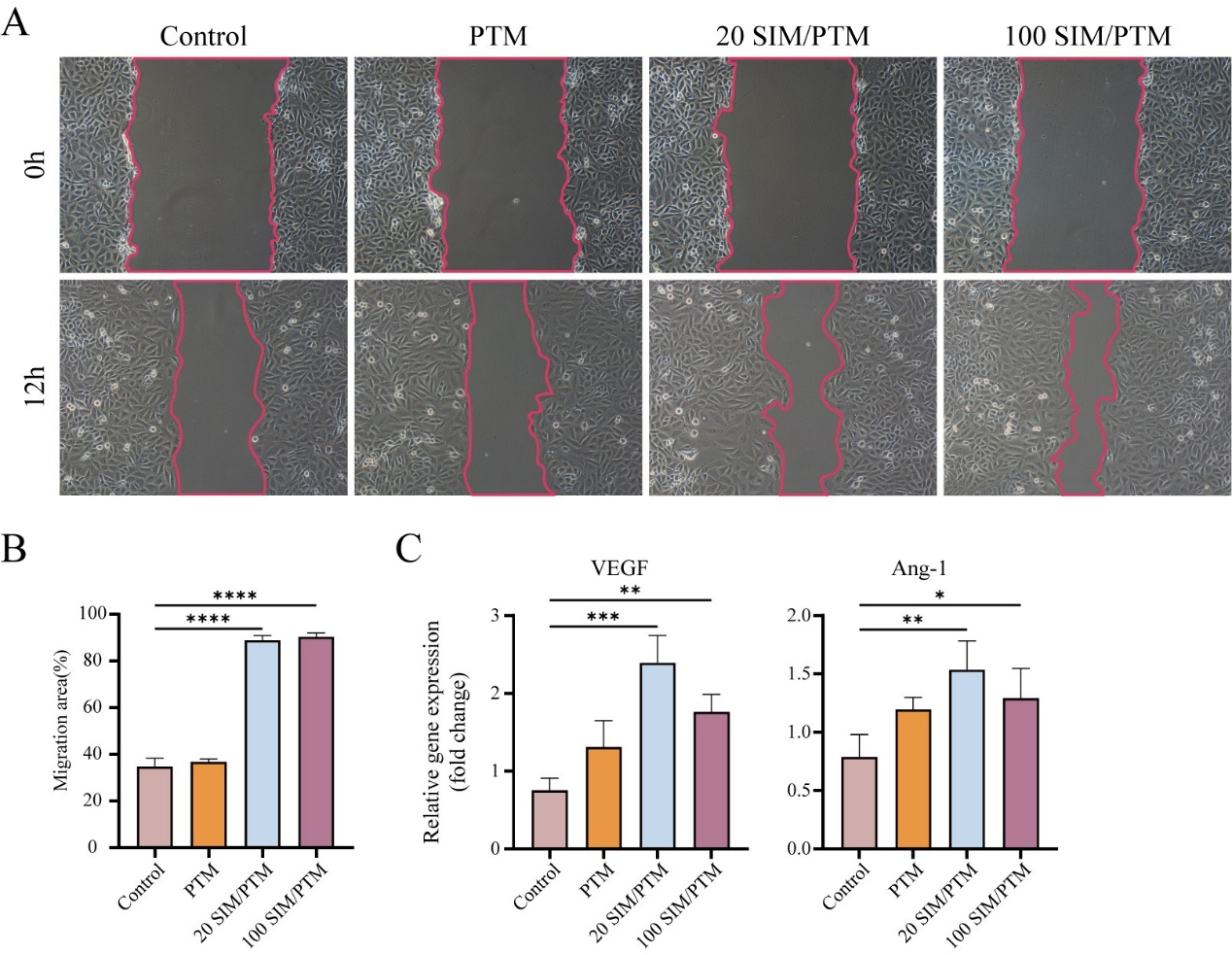


Figure S9 (A) Scratch test (B) Scratch test quantitative analysis (C) PCR analysis of vascular endothelial cell angiogenesis indicators


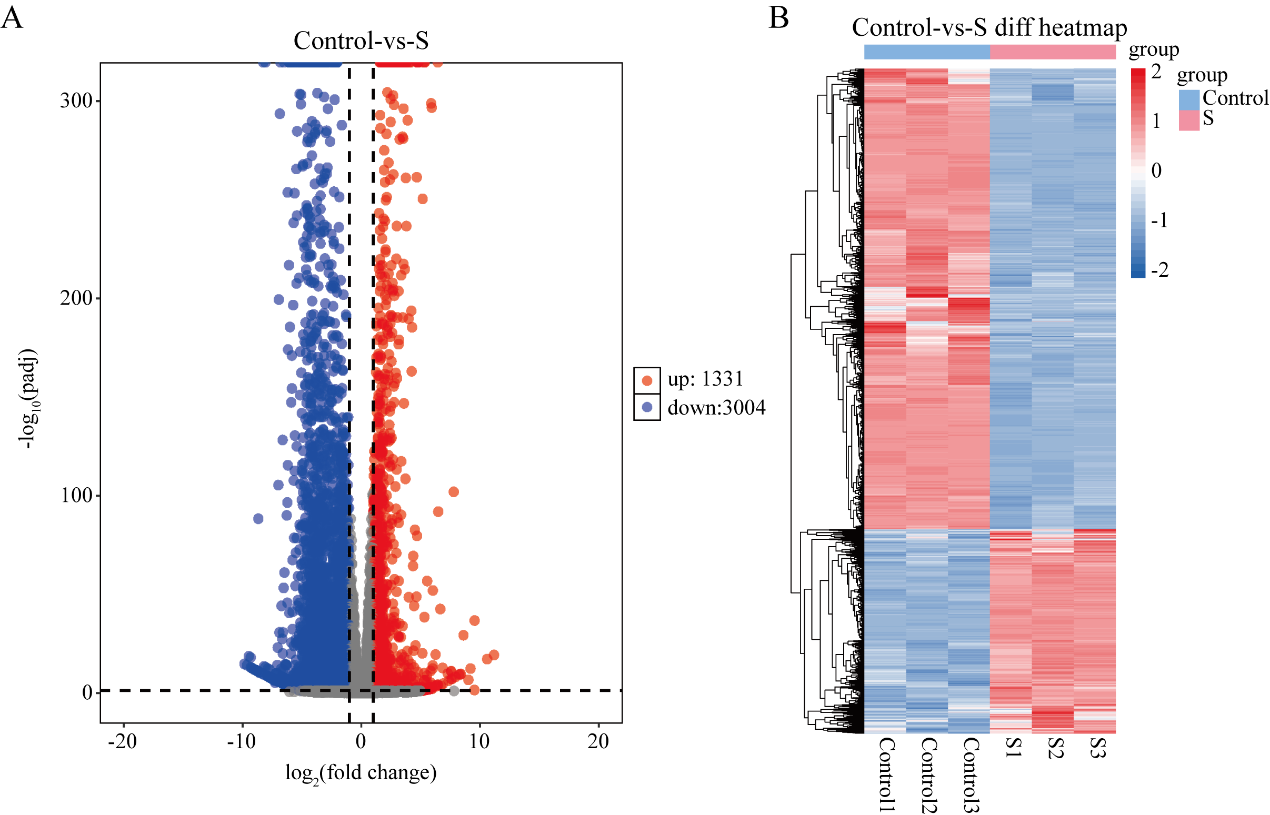


Figure S10 (A) Migration gene volcano map (B) Heat map of migrating genes


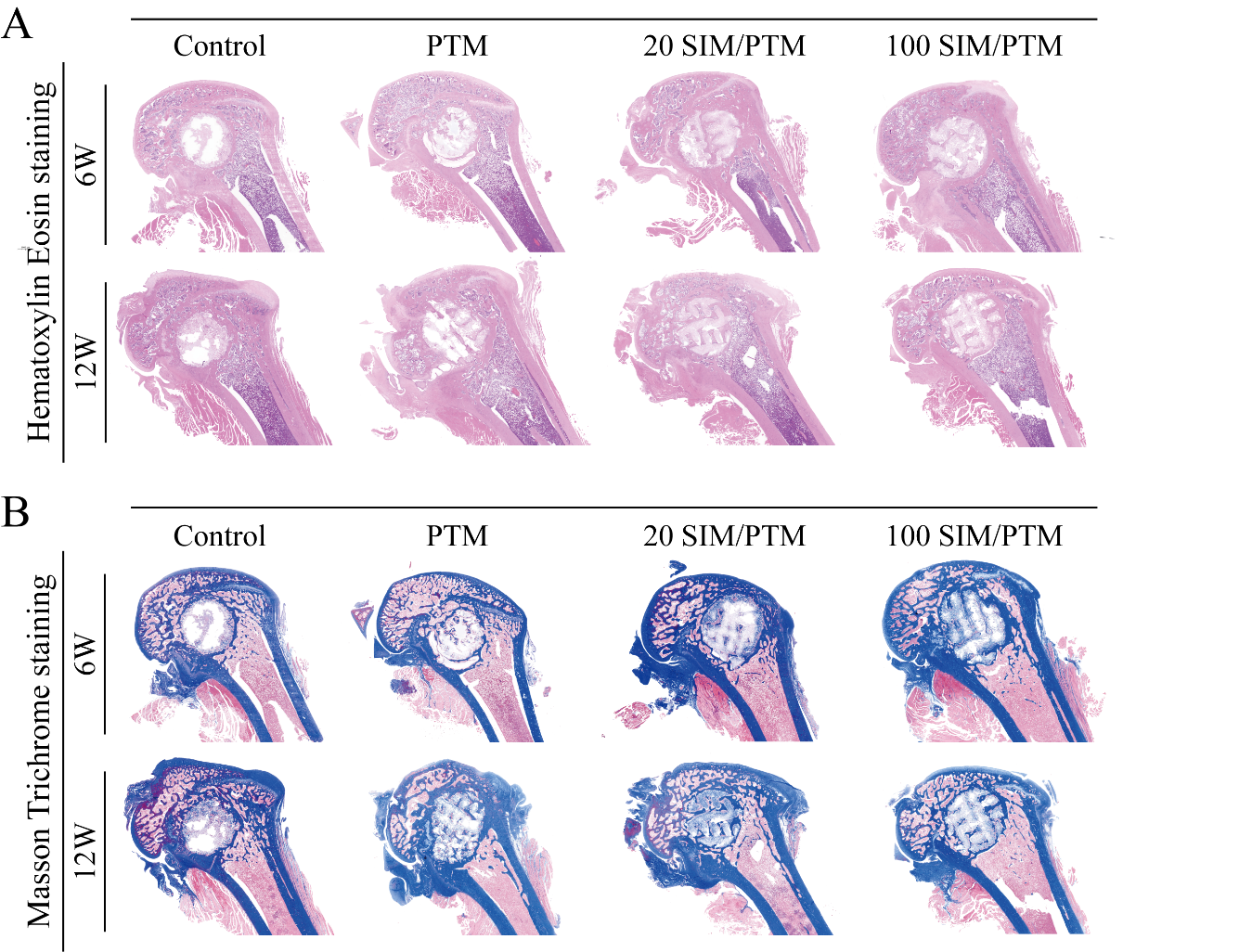


Figure S11 Histological analysis of the defect by H&E and Masson trichrome staining.
